# Into the Fourth Dimension: Dysregulation of Genome Architecture in Aging and Alzheimer’s Disease

**DOI:** 10.3389/fnmol.2018.00060

**Published:** 2018-02-28

**Authors:** Warren Winick-Ng, R. Jane Rylett

**Affiliations:** ^1^Department of Physiology and Pharmacology, Schulich School of Medicine & Dentistry, University of Western Ontario, London, ON, Canada; ^2^Molecular Medicine Research Laboratories, Robarts Research Institute, University of Western Ontario, London, ON, Canada

**Keywords:** Alzheimer’s disease, epigenetics, gene regulation, genome architecture, protein mislocalization

## Abstract

Alzheimer’s disease (AD) is a progressive neurodegenerative disease characterized by synapse dysfunction and cognitive impairment. Understanding the development and progression of AD is challenging, as the disease is highly complex and multifactorial. Both environmental and genetic factors play a role in AD pathogenesis, highlighted by observations of complex DNA modifications at the single gene level, and by new evidence that also implicates changes in genome architecture in AD patients. The four-dimensional structure of chromatin in space and time is essential for context-dependent regulation of gene expression in post-mitotic neurons. Dysregulation of epigenetic processes have been observed in the aging brain and in patients with AD, though there is not yet agreement on the impact of these changes on transcription. New evidence shows that proteins involved in genome organization have altered expression and localization in the AD brain, suggesting that the genomic landscape may play a critical role in the development of AD. This review discusses the role of the chromatin organizers and epigenetic modifiers in post-mitotic cells, the aging brain, and in the development and progression of AD. How these new insights can be used to help determine disease risk and inform treatment strategies will also be discussed.

## Introduction

Non-dividing post-mitotic cells present a unique challenge in biology—how can cells survive, in some cases for the entire lifespan of an organism, while dealing with environmental challenges such as oxidative or endoplasmic reticulum stress, DNA damage, or changes in metabolic state? Many cellular homeostatic changes require a DNA response to either activate or repress transcription of response genes, which implies state-dependent dynamic regulation of gene access. Thus, it is thus critical for the cell to have tools dedicated to facilitating these responses.

The regulatory control systems that dynamically alter transcriptional responses of the DNA as a function of cell state are often understood as the “epigenome”. Examples of well-known histone modifications include methylation and acetylation, but can also include phosphorylation, ubiquitination and sumoylation. Non-histone changes to chromatin are also possible. For example, direct modification of nucleosome position can change which portion of the DNA is accessible to the transcription machinery, while methylation of DNA can restrict access of transcriptional activators. Taken together, chromatin modifications represent a complex system that dynamically regulates gene expression (Duan and Blau, 2012; Fraser et al., 2015). Another level of chromatin regulation results from the linear and spatial organization of genes into functional regions. In fact, gene expression depends on the four-dimensional folding of chromatin in space and time, where active and inactive segments of the genome tend to segregate in the same co-regulated space (Dixon et al., 2012). Higher-order chromatin structures dynamically adapt to nuclear function and complexity in specific cell types and environmental conditions (Duan and Blau, 2012; Fraser et al., 2015; Boettiger et al., 2016).

The four-dimensional folding of chromatin is essential for neuronal development and function, as proper genome architecture is imperative for promoting accurate context-dependent epigenetic modifications. Defects in chromatin-associated processes have been implicated in many neurological disorders, such as schizophrenia, intellectual disorder and autism spectrum disorder (Gong et al., 2008; Gregor et al., 2013; McCarthy et al., 2014). Changes in chromatin topology have also been observed in cultured cells expressing proteins having mutations found in patients with Alzheimer’s disease (AD; Walker et al., 2013), a progressive neurodegenerative disease characterized by synaptic dysfunction of cholinergic and other neurons, memory loss and cognitive deficits (Sheridan and Hausdorff, 2007; McCade et al., 2011; Arshavsky, 2014).

AD represents a large global health care burden, with 47 million individuals world-wide living with AD-related dementia at an annual cost of US$ 818 billion (or ~1% of the global gross domestic product) in 2016; this is estimated to reach $1 trillion in 2018 (World Alzheimer Report, 2015, 2016). With the incidence of AD expected to climb to 75 million by 2030, the estimated annual cost is expected to reach US$ 2 trillion at that time (World Alzheimer Report, 2015). The progressive dysfunction and eventual loss of cholinergic neurons characterize AD, resulting in impairments in memory, cognition, attention, mood and motor control (for reviews see Sheridan and Hausdorff, 2007; McCade et al., 2011; Arshavsky, 2014; Stella et al., 2014). The development and progression of pathology associated with AD is complex and not well understood. While AD is the most common form of dementia, only ~1%–5% of cases can be explained completely by genetic origin (Piaceri et al., 2013); this is most commonly related to mutations in amyloid precursor protein (APP) or presenilin 1/2 (PSEN1/PSEN2) that result in the overproduction of toxic β-amyloid peptides (Aβ) and an early-onset of disease symptoms (for reviews see Tanzi, 2012; Wu et al., 2012). Accumulation of Aβ together with hyper-phosphorylation of the microtubule-associated protein Tau are hypothesized to underlie reduced synaptic transmission, neuronal dysfunction and neurodegeneration in AD (Alonso et al., 1996; reviewed in Hardy and Selkoe, 2002; Zussy et al., 2013), though the etiology of the ~95% of “sporadic” late-onset cases is unclear.

Miller et al. (2008) have provided evidence that individuals with moderate to severe AD have large changes in transcription for functional groups of genes related to ion transport, synaptic transmission and RNA processing, among others. Despite large-scale transcriptional changes, efforts to identify susceptibility genes have been largely unsuccessful; only a handful of genes have been identified that have single nucleotide polymorphisms (SNPs) or alleles related to AD susceptibility (Bertram, 2011; Lambert et al., 2013; Piaceri et al., 2013). Further, Norton et al. (2014) estimated that up to a third of AD cases may be attributed to modifiable environmental risk factors, including level of physical activity, smoking and obesity, among others. Together, these data suggest that both genetic and environmental factors may underlie the observed transcriptional changes in sporadic AD, providing rationale for a recent focus on identifying whether epigenetic changes also occur in AD and related dementias. However, there is conflicting evidence on the extent and direction of the resulting changes to transcription which may be explained by new evidence implicating changes to organizers of genome architecture and chromatin modifiers in the AD brain (Mastroeni et al., 2013, 2015; Lu et al., 2014; Sen et al., 2015; Winick-Ng et al., 2016). Therefore, understanding how genome architecture can become disrupted in AD is critical for understanding the etiology and progression of AD, as well as for the development of novel therapeutics. This review addresses how chromatin is organized in post-mitotic cells, and the disruptions in genome architecture observed in aging and neurodegenerative disease. This review also addresses how understanding chromatin topology and changes to chromatin organization may inform future therapeutic targets in neurodegenerative disease.

## Chromatin Organization in Post-Mitotic Cells

In non-dividing cells the genome is generally organized into two transcriptional states: euchromatin—DNA that is available for transcription and loosely packed; or heterochromatin—highly compacted DNA that is often transcriptionally repressed (Croft et al., 1999; Zink et al., 2004; Harnicarová et al., 2006). Heterochromatin can be further divided into facultative repression—DNA that is transiently repressed and easily reversed; and constitutive repression—a more permanent repressive state (Li and Zhou, 2013; Saksouk et al., 2015; Stunnenberg et al., 2015; Jamieson et al., 2016). Heterochromatin is found largely at the nuclear periphery, associated with nuclear lamina proteins, lamin A or B, or in peri-nucleolar regions (Peric-Hupkes et al., 2010; Shah et al., 2013; reviewed in Luperchio et al., 2014). Euchromatic regions of activated chromatin are mainly found in the nuclear interior, with very little nuclear membrane or lamina association (for reviews see Kalverda et al., 2008; Luperchio et al., 2014). Euchromatic and heterochromatic regions are influenced by, and can directly result from, the dynamic interplay of epigenetic modifications to histones and DNA (for reviews see Sharma et al., 2010; Politz et al., 2013; Alexander and Lomvardas, 2014; Luperchio et al., 2014).

In the absence of external influences, post-mitotic cells must maintain proper nuclear organization for their entire lifespan. In contrast, a mitotic cell changes its chromatin organization during cell division, as decondensed interphase chromosomes must become compacted during prophase and remain in this state until cell division is complete, before re-establishing their configuration when nuclei reform (Hinde et al., 2012). An advantage to having a localization-dependent organization of the genome is that cells can modulate the extent of gene activation and response time to environmental input by altering the localization of individual genes. One example of organization-dependent gene activity in neurons is for the brain-derived neurotropic factor (BDNF) gene. *BDNF* encodes BDNF, a protein important for neuron development, survival and synaptic development (Bamji et al., 2006; Chen et al., 2013). In addition, BDNF signaling in hippocampal neurons mediates increases in dendritic spine density during chemically-induced long-term potentiation (Montalbano et al., 2013). In adult non-dividing neurons, *BDNF* is localized to the nuclear periphery. However, after repeated activation of hippocampal neurons by kainate-induced seizures, the gene is rapidly internalized and activated (Walczak et al., 2013). *Bdnf* mRNA levels peak by 2 h after stimulation, with this fully attenuated by 7 days. Though the mRNA levels are no longer elevated, an interesting observation is that the gene remains localized in the nuclear interior for at least 28 days following stimulation (Walczak et al., 2013). The *BDNF* localization change is important for sensitivity to the next stimulation. When Walczak et al. (2013) stimulated these cells 28 days following the initial stimulation, RNA polymerase II (Pol II) recruitment to the *BDNF* gene and *Bdnf* mRNA levels were significantly increased compared to cultured cells from naïve animals, suggesting that gene localization is important for magnitude of response.

There are a few exceptions to the peri-nucleolar and lamina-associated heterochromatin observed in neurons. Olfactory neurons, which each have a uniquely expressed olfactory receptor (OR), have a large amount of heterochromatin in the center of the nucleus due to developmental downregulation of the lamin B receptor that normally helps anchor heterochromatin to the nuclear periphery (Clowney et al., 2012). This repressive heterochromatin “core” contains the OR genes, which are enriched with repressive histone markers and constitutively repressed (Clowney et al., 2011, 2012; Magklara et al., 2011). During development, one of the ~2800 repressed OR genes is looped out of the core, and associated with a distal transcription enhancer leading to gene activation (Lomvardas et al., 2006; Clowney et al., 2012). Additionally, exceptions in heterochromatin organization occur in retinal rod cells of nocturnal animals. At birth, nocturnal animals display a typical pattern of chromatin organization, but by post-natal day 14 (P14) nuclear lamina proteins are downregulated leading to the internalization of the heterochromatin (Solovei et al., 2013; reviewed in Alexander and Lomvardas, 2014). By adulthood, and throughout the entire lifespan of these animals, heterochromatin is found exclusively in the center of the nucleus with the euchromatin surrounding it (Solovei et al., 2009, 2013). This inside out configuration allows for the rod cells to diffract light more efficiently at night, when these cells are more active (Solovei et al., 2009).

## Four-Dimensional Chromatin Organization in Post-Mitotic Cells

**Figure 1:**
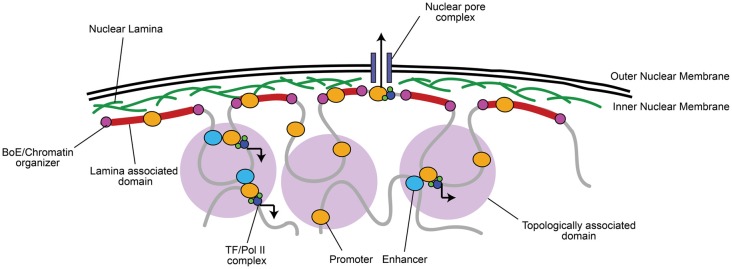
Chromatin is organized into topologically and lamina associated domains (LADs). Chromatin is organized into large 0.1–1 megabase pair (Mb) wide topologically-associated domains (TADs), flanked by boundary elements (BoEs) that organize chromatin and allow access for chromatin modifying proteins (Dixon et al., 2012; Heidari et al., 2014; Fraser et al., 2015). Three-dimensional chromatin looping within TADs can bring non-coding regulatory elements (e.g., enhancers) to promoters, which can recruit transcriptional machinery, such as RNA Polymerase II (Pol II) and transcription factors (TFs; Dekker et al., 2002; Simonis et al., 2007; Sanyal et al., 2012). TADs found at the nuclear periphery are associated with lamin A/B, often constitutively repressed, and termed LADs (Guelen et al., 2008). A notable exception is where euchromatic regions of LADs interact with nuclear pore complexes for rapid mRNA transport to the cytoplasm (Brickner et al., 2012).

There is growing agreement that chromatin organization is more complex than simple heterochromatin/euchromatin regions. Non-coding regions, which represent approximately 98% of the genome, contain key regulatory information. Non-coding elements (e.g., enhancers) control gene activation by establishing physical contacts with genes in three-dimensional space (Figure 1; Duan and Blau, 2012; Fraser et al., 2015; Pombo and Dillon, 2015; Boettiger et al., 2016). Importantly, long-range contacts between enhancers and promoters are thought to recruit Pol II and transcription factors (TFs) for transcriptional activation (Figure 1; Dekker et al., 2002; Simonis et al., 2007; Sanyal et al., 2012).

Active and inactive segments of the genome tend to segregate in space, while co-regulated regions group within the same topologically associating domain (TAD), which are thought to contribute to their coordinated expression or silencing (Dixon et al., 2012; Heidari et al., 2014; reviewed in Luperchio et al., 2014; Fraser et al., 2015; Figure 1). Centrally localized TADs tend to be enriched in histone H3 lysine 27 tri-methylation (H3K27me3), a histone modification marking facultative repression, and are flanked by boundary elements (BoEs); DNA/protein complexes (also termed insulators) that can organize chromatin into looped structures (Lin et al., 2011; Dixon et al., 2012; Heidari et al., 2014). BoEs allow DNA to be accessed by chromatin remodeling proteins, TFs, and Pol II, and are enriched with H3K27me3 or H3K4me3, a marker of active transcription, depending on the state of transcriptional activation (Dixon et al., 2012; Narendra et al., 2015). TAD architecture has been described as a domain within domain system: inter-TAD contact results in coordinated regulation of inter-TAD regions into megabase pair (Mb) large “meta-TADs” (Heidari et al., 2014; Fraser et al., 2015), and BoEs can also be found within 50–100 kb “sub-TAD” regions (Dixon et al., 2012; Heidari et al., 2014). Meta-TAD and sub-TAD interacting regions are often sites of transcriptional activation (Heidari et al., 2014; Fraser et al., 2015; Smith et al., 2016). TADs can also interact with and bind to the nuclear lamina at 0.1–10 Mb wide lamina-associated domains (LADs; Guelen et al., 2008). The finding of LADs correlates well with observations of peripheral heterochromatin, as LADs are mainly enriched with Histone 3 lysine 9 tri-methylation (H3K9me3), a marker of constitutive repression, and highly compacted (Guelen et al., 2008; Meuleman et al., 2013). LADs are also flanked by BoEs that interact with chromatin enriched with H3K27me2/3 and accessible to chromatin organizers (Guelen et al., 2008), which is critical to maintain transcriptional repression but still allow access to genes when environmental state-dependent changes occur. As an exception, genes within LADs that are localized near nuclear pores are often euchromatic to facilitate transcriptional activation and rapid mRNA transport out of the nucleus (Brickner et al., 2012).

Chromatin organization in post-mitotic cells is critical for neuronal development and synaptic plasticity. For example, *CTCF* encodes CTCF (CCCTC-binding factor), a BoE critical for chromatin looping and genome organization (Dixon et al., 2012). Children born with mutations or deletions in *CTCF* display intellectual disability, microcephaly and often show autistic features (Gregor et al., 2013), and *CTCF* was also identified recently as a schizophrenia susceptibility gene (Juraeva et al., 2014). Mutations in cohesin, a protein that works together with CTCF to spatially organize chromatin loops (Busslinger et al., 2017; Hanssen et al., 2017), causes Cornelia de Lange syndrome (Musio et al., 2006). Fujita et al. (2017) found that depletion of cohesion in the cerebral cortex of mice leads to the disruption of synapse formation and heightened anxiety to novel environments. Further, dysregulation of Alpha Thalassemia/Mental Retardation Syndrome X-Linked (ATRX), a protein involved in chromatin remodeling and downregulation of gene expression, has also been linked to intellectual disorder and autism-spectrum disorder (Gong et al., 2008; Martínez et al., 2014).

In terms of normal neural development and synaptic plasticity, Vogel-Ciernia et al. (2013) showed that the Brg1-associated factor subunit BAF53b (part of the SWI/SNF chromatin remodeling complex) is important for long-term memory consolidation, dendritic arborization and hippocampal synaptic plasticity. Mutations in BAF53b leads to impairments in synaptic plasticity, driven by abnormal gene expression of postsynaptic genes (Vogel-Ciernia et al., 2013). Thus, chromatin organization is important for proper neuron function and development, but may also be important for environmental and context-based gene regulation.

## Regulation of Genome Architecture at Scaffolding/Matrix Attachment Regions

**Figure 2:**
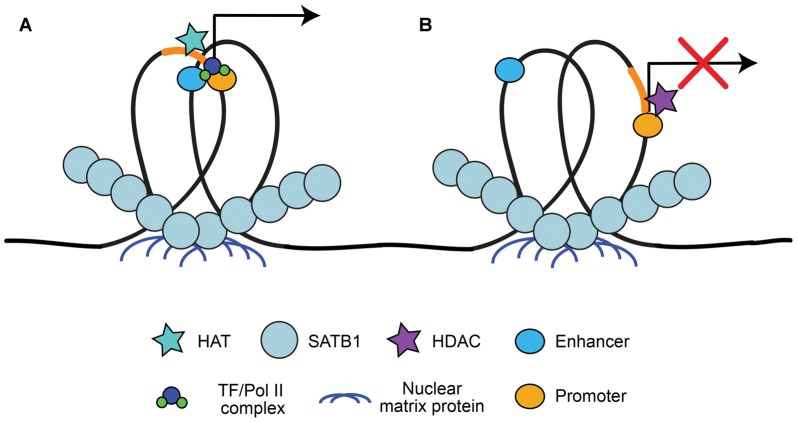
Special AT-rich binding protein 1 (SATB1) recruitment to scaffolding/matrix attachment regions (S/MARs). SATB1 binds to DNA at regions that interact with the nuclear matrix, known as S/MARs. This results in locus specific chromatin looping of local or distant genes that can be accessed by chromatin organizers. **(A)** SATB1-mediated looping at S/MARs can bring distal enhancers close to gene promoters and recruit histone acetyltransferase (HATs) for acetylation, resulting in RNA Pol II/TF recruitment and transcriptional activation (Wen et al., 2005; Wang et al., 2009; Padmaja et al., 2010; Mirlekar et al., 2017). **(B)** SATB1 can also segregate enhancers from promoters and recruit histone deacetylases (HDACs), resulting in facultative or constitutive repression (Padmaja et al., 2010; Kohwi-Shigematsu et al., 2013; Mirlekar et al., 2017).

A large advantage of having a high level of organization of chromatin is the ability to have long-range state-dependent dynamic regulation of chromatin access, accomplished by accessing the chromatin loops created by chromatin organizers for locus-specific regulation. One method to produce state-dependent looping involves anchoring either local or distant genes to nuclear matrix proteins at scaffolding/matrix attachment regions (S/MARs; Heng et al., 2004; reviewed in Politz et al., 2013). DNA is anchored to S/MARs by organizers such as special AT-rich binding protein 1 (SATB1; Cai et al., 2003; reviewed by Kohwi-Shigematsu et al., 2013) and S/MAR-binding protein 1 (SMAR1; Sinha et al., 2010). S/MAR organizers recruit chromatin modifying enzymes to the region to either activate or repress transcription, depending on what is recruited (Figure 2). For example, SATB1 can recruit the histone deacetylase (HDAC) HDAC1 to repressed regions and the histone acetyltransferase (HAT) p300 to activated regions (reviewed by Kohwi-Shigematsu et al., 2013). SATB1 has been shown previously to be important for anchoring chromatin loops on the beta-globin locus to bring the gene encoding the activated beta-globin subunit close to hypersensitive sites (i.e., enhancers), but segregate other genes away from this region (Wen et al., 2005; Wang et al., 2009). Indeed, S/MAR formation may help organize promoter-enhancer contacts, and dynamically regulate promoter activation (Figure 2; Padmaja et al., 2010; Mirlekar et al., 2017). SATB1-mediated chromatin looping is also involved in cytokine activation through the TH2-cytokine (IL4/5/13) locus in T-cells (Cai et al., 2006), regulation of the switch from the pro-apoptotic *BCL2* gene to the anti-apoptotic *NOXA* gene (Yang et al., 2015), X-chromosome inactivation (Agrelo et al., 2009), as well as in regulating gene expression of the major histocompatibility complex (MHC) class I locus (Kumar et al., 2007).

S/MARs may also be involved in context-dependent regulatory events. In HCT116 p53+/+ cells exposed to UV radiation to induce DNA double strand breaks, SMAR1 recruits HDAC1 to the *BAX* and *PUMA* promoters, which results in deacetylation and repression of these pro-apoptotic genes (Sinha et al., 2010). However, if DNA damage is extensive, SMAR1 releases HDAC1, allowing acetylation of the *BAX* and *PUMA* promoters and the initiation of apoptosis (Sinha et al., 2010). While this is a good example of how cells can respond to environmental challenges, the mechanisms by which state-dependent regulation works and under which conditions cells utilize these processes are still being investigated. There are only a few studies assessing how organizers such as SATB1 or SMAR1 function in neural cells, with this focusing solely on neural development (Balamotis et al., 2012; Close et al., 2012; Wang et al., 2015). However, Jaitner et al. (2016) showed recently that SATB2, which has a similar function to SATB1 and is highly expressed in CA1 hippocampal neurons, is critical for long-term synaptic plasticity as well as long-term fear and object recognition memory. It is imperative to understand how chromatin organization may contribute to cell-state changes, as genome architecture is disrupted in aging cells and in neurodegenerative diseases such as AD.

## Dysregulation of Genome Architecture in Aging

Recently, there has been interest in chromatin organization related changes during aging and senescence. Shah et al. (2013) showed that LADs predominantly enriched with H3K9me3 start to lose this histone marker during aging, replaced by H3K27me2/3 “canyons” and H3K4me3 “mesas” in senescent human IMR90 cells. Along with decreased lamin A and B, the increase in H3K4me3 results in abnormal activation of genes and destabilization of peripheral-associated heterochromatin leading to gene internalization (Shah et al., 2013). It has been proposed that aging-related increases in H3K4me3 may be a model for the increases in inappropriate gene activation seen in many cell types during both aging and senescence (reviewed in Luperchio et al., 2014). Further, we can speculate that regions enriched with H3K27me2/3 would be more easily activated compared to H3K9me3 enrichment as repressive H3K27me2/3 modifications are associated with transcriptional pausing (Min et al., 2011), and are more easily reversed compared with H3K9me3 (Sarcinella et al., 2007).

In post-mitotic neurons, aging related changes in epigenetic regulation are less clearly defined. Studies have reported increases in H3K27me2/3, H3K9me3 and DNA methylation, leading to inappropriate repression of gene expression (Wang et al., 2010; Hernandez et al., 2011; Walker et al., 2013). In addition, Cheung et al. (2010) found that in aging human prefrontal cortex neurons there are approximately 100 genes abnormally enriched in H3K4me3, all related to cell cycle regulation. In a separate study from Tang et al. (2011), neurons from human prefrontal cortex of older subjects had reductions in histone acetylation of H3K9/K14 on genes related to inhibitory neurotransmission and mitochondrial function. While the contributions of epigenetic changes to aging-related neural phenotypes remain unknown, senescence-like phenotypes have been observed in cortical neurons following DNA damage (Jurk et al., 2012), and in aging dopaminergic and cholinergic neurons (Panossian et al., 2011). Finally, aging-related downregulation of lamin B in neurons results in centrally located senescence-associated heterochromatic foci (Jurk et al., 2012). Given the link between senescence and the destabilization of the nuclear lamina to inappropriate gene expression changes, understanding the architectural changes to genomic structure in aging neurons will be an important target for future studies.

## Epigenetic Changes in Alzheimer’s Disease

In addition to chromatin-related changes in aging, there are also large scale epigenetic changes in aging-related diseases, such as in AD. Initial studies exploring epigenetic histone modifications in AD have shown complex and variable results (summarized in Table 1). For example, CK-p25 AD model mice have abnormal enrichment of H3K4me3 and H3K27 acetylation (H3K27ac) on promoters of immune response genes (Gjoneska et al., 2015), whereas in the same mice neuroplasticity-related genes show hypoacetylation and reduced enrichment of H3K4me3 (Gräff et al., 2012; Gjoneska et al., 2015). Primary cortical neuron cultures treated with Aβ also have increased H3K4me3 on somatostatin and cortistatin genes (Rubio et al., 2012), and in 3xTg AD model mice H3 and H4 acetylation is elevated (Walker et al., 2013). Findings from human post-mortem brain samples are also variable, with some studies reporting increases and others describing decreases in global acetylation levels (Gräff et al., 2012; Zhang et al., 2012; Narayan et al., 2015). The 3xTg AD model mice show increases in H3K9me2 levels and related chromatin compaction (Walker et al., 2013), further adding to the complexity of the epigenetic changes seen in AD-like pathology. In human necropsy brain samples taken from frontal and temporal cortex, Coppieters et al. (2014) observed DNA hypermethylation in neurons, but not in astrocytes or microglia. The complexity of epigenetic changes in AD, as well as differences seen in the murine models, may be due at least in part to gene specific changes and targets in non-coding genomic regions.

**Table 1:** Epigenetic histone modifications observed in Alzheimer’s disease (AD).

Reference	Model system	Observed change	Effect on transcription
Guan et al. (2009)	HDAC2 overexpressing/ knockout mice; HDAC2 inhibition	HDAC2 associated with synaptic plasticity genes, correlated with reduced synapse density and memory loss	↓ on synaptic plasticity genes of HDAC2 overexpressing mice, ↑ on synaptic plasticity genes of HDAC2 knockout mice or after HDAC2 inhibition
Gräff et al. (2012)	CK-p25 mice; Post-mortem tissue from AD patients	Increased HDAC2 levels	↓ on synaptic plasticity genes
Rubio et al. (2012)	Primary cortical cultures—Aβ treated	Enrichment of H3K4me3	↑ on somatostatin and cortistatin genes
Zhang et al. (2012)	Post-mortem tissue from AD patients	Decreased H3K23Ac and H3K18Ac in temporal lobe	Not measured
Walker et al. (2013)	3xTg AD mice	Increased H3 and H4 acetylation, Some regions with increased H3K9me2	↓ *Bdnf* transcription
Gjoneska et al. (2015)	CK-p25 mice	Enrichment or depletion of H3K4me3, H3K27ac	↑ on immune response genes; ↓ on synaptic plasticity genes
Narayan et al. (2015)	Post-mortem tissue from AD patients	Increased total and acetyl H3/H4 in temporal gyrus	Not measured

Non-coding RNA elements, such as micro-RNAs (miRNAs) and long non-coding RNAs (lncRNAs), have been shown increasingly to be altered in AD. miRNAs are small non-coding RNAs (18–25 nucleotides), many of which have been identified as being expressed specifically in the brain, with roles in transcriptional activation and repression (Shao et al., 2010; Babiarz et al., 2011). Several groups have identified miR-132 as a miRNA downregulated in AD patients and associated with AD risk (Cogswell et al., 2008; Lau et al., 2013; Wong et al., 2013). miR-132 is a repressor of FOXO3, a transcriptional regulator of apoptosis (Wong et al., 2013), and targets Tau, resulting in a negative correlation to Tau tangle formation in the prefrontal cortex of AD patients (Lau et al., 2013). Other miRNAs may also have a role in AD (reviewed in Tan et al., 2013), such as miR-34c which is significantly upregulated in APP/PS1 AD model mice and correlated with impaired memory function (Zovoilis et al., 2011). As another example, miR-29a and -29b are significantly reduced in human sporadic AD patient samples (Hébert et al., 2008). The authors found that miR-29a and -29b binds to the β-secretase 1 (BACE1) promoter in HEK-293 cells (Hébert et al., 2008). BACE1 promotes amyloidogenic processing of APP; when Hébert et al. (2008) expressed miR-29a/b transiently in HEK cells that also expressed mutant APP, the levels of BACE1 and Aβ peptide were reduced.

LncRNAs are non-coding RNAs >200 nt in length and localized to the nucleus with essential roles in transcriptional regulation, genome organization, translation and splicing (for reviews see Quinodoz and Guttman, 2014; Angrand et al., 2015). Several LncRNAs are hypothesized to be involved in AD. For example, BACE1 anti-sense transcript (BACE1-AS) is upregulated along with BACE1 in AD model mice, human post-mortem tissue, and following exposure to Aβ in human embryonic kidney (HEK) cells (Faghihi et al., 2008; Modarresi et al., 2011). Knockdown of BACE1-AS in HEK cells or in AD model mice reduces BACE1 mRNA levels and Aβ production, suggesting that both BACE1 and BACE1-AS are necessary for amylodogenic cleavage of APP (Faghihi et al., 2008; Modarresi et al., 2011). The LncRNA 17A causes alternative splicing of the GABA B2 receptor and disrupts the receptor’s intracellular signaling (Massone et al., 2011). 17A is significantly increased in AD patients, but has not yet been linked directly with the mechanisms of the disease (Massone et al., 2011).

Changes in gene expression of some genes in AD may also be directly due to the association of Aβ with DNA (reviewed in Multhaup et al., 2015). Following non-amylodogenic processing, the APP intracellular domain (AICD) forms a complex with Fe65 and Tip60 (von Rotz et al., 2004; Müller et al., 2013). The AICD-Fe65-Tip60 complex translocates to the nucleus, and is thought to regulate the expression of several target genes related to APP-processing (including APP itself), cellular growth, and the cell stress response (von Rotz et al., 2004; Barucker et al., 2014, 2015). Interestingly, Aβ_1–42_ has also been found to associate with some AICD-Fe65-TIP60 target genes in SH-SY5Y cells by ChIP (Barucker et al., 2014). Giuffrida et al. (2009) showed that Aβ monomers activated the phosphatidylinositol-3-kinase pathway in rodent cortical cultures, promoting neuronal survival. Others have shown that the more toxic oligomeric Aβ can induce re-entry into the cell cycle and abnormal DNA replication, leading to apoptosis (Copani et al., 2006; Varvel et al., 2008).

Importantly, numerous studies that show gene specific modifications of histone deactylation in AD cell and rodent models, as well as in humans. For example, in aged AD model mice and human AD patients have increased HDAC2-related deacetylation of synaptic plasticity related genes (Guan et al., 2009; Gräff et al., 2012). Hu et al. (2017) showed that apicidin-mediated inhibition of HDACs in human SH-SY5Y cells increased the expression of a disintegrin and metalloproteinase domain-containing protein 10 (ADAM10). ADAM10 promotes non-amyloidogenic processing of APP and is thought to be neuroprotective (reviewed by Endres and Fahrenholz, 2012). Volmar et al. (2017) showed that the class I/II HDAC inhibitor M344 increased ADAM10 in 3xTg mice, along with several other changes in expression of APP processing related genes. Further, overexpression of HDAC3 in the hippocampus of AD model mice increases Aβ levels and decreases dendritic spine density (Zhu et al., 2017), and multiple studies report that inhibition of HDACs in AD mouse models improves learning and memory deficits (Guan et al., 2009; Rumbaugh et al., 2015; Volmar et al., 2017; Zhu et al., 2017). While these results are promising, the specificity of the gene targets and target regions highlight the difficulty in finding effective treatments (reviewed in Yang et al., 2017). It is clear that more studies are needed to help define the complex epigenetic landscape in aging and AD, especially as models for sporadic AD are not well developed and findings *in vitro* or in animals may not fully reflect changes observed in human patients.

## Chromatin Organizers and Epigenetic Regulators Are Mislocalized in Alzheimer’s Disease

Epigenetic changes in AD are highly variable and likely gene specific, which suggests possible changes to non-coding regions and to chromatin organization. In support of this hypothesis, multiple reports suggest that chromatin organizing proteins may be linked to AD etiology. For example, shRNA depletion of chromodomain helicase DNA binding protein 5 (CDH5, a paralog to the mi-2/Nurd remodeling complex) in primary cultures of rat brain cortex can cause both activation and repression of aging and AD-related genes (Potts et al., 2011). Interestingly, Zhang et al. (2009) showed that inhibiting SATB1 orthologs in *C*.* elegans* reduces lifespan, and further show that changes in SATB1 expression levels and activation may also be related to AD pathology; knockdown of CREB-binding protein, a binding partner for SATB1, accelerates Aβ-induced paralysis in a transgenic *C*.* elegans* AD model (Zhang et al., 2009). In PS19 AD model mice, there are observed reductions in sirtuin 1 (SIRT1), a histone and protein deacetylase that activates SATB1 (Xue et al., 2012; Cho et al., 2015). SIRT1 expression increases activation of ADAM10 in human glioma cells and N2a cells (Theendakara et al., 2013; Lee et al., 2014). Interestingly, Hernandez-Rapp et al. (2016) showed that miR-132 also represses SIRT1, and that both SIRT1 and miR-132 are negatively correlated with Aβ levels in 3xTg AD mice. Given that SIRT1 can deacetylate and activate SATB1 (Xue et al., 2012), reductions in SIRT1 could further affect global chromatin organization, but this remains to be elucidated.

Phosphorylated Tau associated with neurodegenerative pathology may also have a direct effect on chromatin organization in AD. While Tau is most well-known for the stabilization of microtubules, several studies have shown that Tau is also localized to the nucleus in heterochromatic regions, most notably at nucleolar organizing regions (Loomis et al., 1990; Sjöberg et al., 2006; Rossi et al., 2008). The role of Tau at these regions is likely related to mitotic progression, expression of an adult form of Tau during *Drosophila* development leads to delayed mitotic progression in neural progenitors, resulting in aneuploidy in post-mitotic cells and reduced lifespan (Malmanche et al., 2017). Of interest, Rossi et al. (2008) found that frontotemporal dementia patients expressing mutant Tau had chromosome abnormalities in fibroblasts and lymphocytes; these included metaphase chromatid breaks, abnormal metaphase chromatin threads, chromosome deletions and decondensing of the prophase nucleus. Several studies now directly link mutations in Tau to chromatin re-organization and neurodegeneration. In *Drosophila* expressing disease-associated mutant Tau, Frost et al. (2014, 2016) observed reductions in lamin B, invaginations of the nuclear envelope, and reductions in heterochromatin markers such as H3K9me2 and heterochromatin protein 1α (HP1α). Importantly, these authors also found nuclear envelope invaginations and reductions in lamin B in neurons in the frontal cortex of human AD post-mortem biopsies (Frost et al., 2016). It is clear that there is now emerging evidence that chromatin organization may be altered in AD, though the extent of these alterations and impact on gene expression require further study.

Recent observations made on neurons in necropsy brain samples from AD patients have revealed that proteins involved in epigenetic regulation and chromatin organization are mislocalized in AD (Table 2). Mastroeni et al. (2013) showed that DNA methyltransferase 1 (DNMT1) and Pol II were abnormally sequestered in the cytoplasm of CA1 hippocampal neurons from AD patients, and were able to recapitulate this effect in human SK-N-Be(2) neuroblastoma cells exposed to toxic Aβ oligomers for 36 h. As another example, repressor element 1-silencing transcription (REST), a neuronal gene repressor that is activated in aging and is involved in the cell stress response, is abnormally localized to autophagosomes in prefrontal cortex neurons of AD patients (Lu et al., 2014). Sen et al. (2015) showed that apolipoprotein E4 (ApoE4), the protein product of the *APOE* ε4 risk allele, increases nuclear translocation of HDAC4 and HDAC6 in SH-SY5Y cells and human primary neurons, resulting in reduced *BDNF* gene expression. In addition, cytoplasmic accumulation of H3K4me3 is observed in neurons from early stage AD patients (Mastroeni et al., 2015).

**Table 2:** Mislocalized proteins in AD and effects on transcription.

Reference	Model system	Observed change	Effect on transcription
Gill et al. (2007)	Post-mortem tissue from MCI and AD patients	Cytoplasmic sequestering of 82-kDa ChAT	Not measured
Mastroeni et al. (2013)	SK-N-BE(2) cells—Aβ treated; Post-mortem tissue from AD patients	Cytoplasmic sequestering of DNMT1 and RNA Pol II	↓ RAN, RANBP1, RANBP2, RANBP5, RNA Pol II transcription
Lu et al. (2014)	Post-mortem tissue from AD patients; REST knockout mice; *C*.* elegans* with *spr-4* mutations	REST localized to autophagosomes and decreased H3K9Ac in AD patients; cell death in knockout mice; decreased survival in *C*.* elegans* mutants	↓ on neurotransmission related genes (late AD only); ↑ on neurotransmission related genes (early AD only), pro-apoptotic genes (early+late AD)
Mastroeni et al. (2015)	Post-mortem tissue from AD patients; 3xTg AD mice	Cytoplasmic sequestering of H3K4me3	Not measured
Sen et al. (2015)	SH-SY5Y cells/Human primary neurons—Aβ and ApoE4 treated cells	HDAC4 and HDAC6 nuclear import	↓ *BDNF* gene expression
Winick-Ng et al. (2016)	SH-SY5Y cells—Aβ treated	82-kDa ChAT and SATB1 localization to S/MARs at synapse-related genes	↓ *APP* mRNA isoform

A human and primate specific 82-kDa variant of choline acetyltransferase (82-kDa ChAT) was also found to have altered localization in cholinergic neurons of patients diagnosed with mild cognitive impairment (MCI) and AD. In healthy young adults, 82-kDa ChAT is localized predominantly in the nucleus of cholinergic neurons, but is mislocalized largely to the cytoplasm in elderly individuals and in patients with MCI and AD (Resendes et al., 1999; Gill et al., 2003, 2007). While the mechanisms regulating the nuclear localization of 82-kDa ChAT have not yet been elucidated, there is now evidence to suggest that this enzyme also plays a role in changes to gene expression and genome architecture (Albers et al., 2014; Winick-Ng et al., 2016). For example, primary neuron cultures prepared from brains of APP/PSEN1 double transgenic AD model mice that are transduced to transiently express 82-kDa ChAT have increased gene expression of golgi-associated, gamma-adaptin ear-containing, ARF binding protein 3 (*GGA3*), which encodes GGA3 (Albers et al., 2014). GGA3 is involved in endosomal trafficking of BACE1 for lysosomal degradation and recycling (Tesco et al., 2007; Kang et al., 2010), and in this model 82-kDa ChAT expression resulted in reductions in the protein levels and activity of BACE1 (Albers et al., 2014). As a result, Albers et al. (2014) showed that there was also decreased production and secretion of Aβ_1–42_ from cultured neurons from brains of APP/PS1 mice expressing 82-kDa ChAT.

82-kDa ChAT interacts with chromatin at S/MARs, co-localizing with SATB1 after differentiated SH-SY5Y cells are exposed acutely to oligomeric Aβ_1–42_ (Winick-Ng et al., 2016). Importantly, chromatin immunoprecipitation-sequencing experiments (Winick-Ng et al., 2016) revealed interactions between 82-kDa ChAT, SATB1 and genes related to synapse function, as well as many genes previously identified in genome-wide association studies (GWAS) meta-analyses as candidate AD-risk genes, such as *BIN1*, *CR1*, *EFNA5*, *MAGI2*, *MTHFD1L* and* PRUNE2* (Bertram, 2011). *GAB2* was another GWAS-identified risk gene identified in this study (Winick-Ng et al., 2016), which encodes GAB2 (GRB2-associated-binding protein 2) that interacts with many receptor tyrosine kinases and membrane proteins such as APP (reviewed in Pan et al., 2010), and has 10 SNPs associated with AD in apolipoprotein E (*APOE*) ε4 carriers (Reiman et al., 2007). Whether the binding of 82-kDa ChAT and SATB1 at S/MARs alters gene expression at these regions, and the consequences for the neuron when 82-kDa ChAT is mislocalized in AD remains to be explored.

## Alzheimer’s as A Disease of Chromatin Organization— Determining Disease Risk and Treatment Strategies

**Figure 3:**
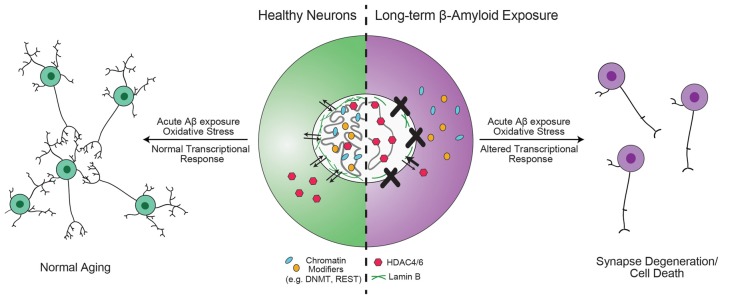
Hypothesis for the role of chromatin organizers in the progression of Alzheimer’s disease (AD). Proteins involved in chromatin organization and topology, such as DNA methyltransferase 1 (DNMT1), repressor element 1-silencing transcription (REST) and 82-kDa choline acetyltransferase (82-kDa ChAT) are critical for the transcriptional response to acute, short-term β-amyloid peptides (Aβ)-exposure (Mastroeni et al., 2013; Lu et al., 2014; Winick-Ng et al., 2016). However, in mild cognitive impairment (MCI) and AD, these proteins are less localized to the nucleus (Gill et al., 2007; Mastroeni et al., 2013; Lu et al., 2014). Other epigenetic modifiers, such as HDAC4 and HDAC6 have increased localization in the nucleus (Sen et al., 2015). In addition, mutant tau expression leads to reduction in lamin B and loss of heterochromatin (Frost et al., 2016). The redistribution of DNMT1 may be due to long-term Aβ-exposure (Mastroeni et al., 2013), but is unknown for other proteins. As a result of mislocalization of REST, neurons are unable to respond appropriately to oxidative or Aβ stress, leading to aberrant transcription that results in synapse degeneration and cell death (Lu et al., 2014).

Gene-specific changes in epigenetic modifications have been observed in AD patients, along with altered localization of critical chromatin modifiers. Together, these data strongly support a hypothesis where the underlying phenotype of sporadic AD can be described as resulting from the failure to produce proper genome architecture and epigenetic modifications. An interesting observation made by Winick-Ng et al. (2016) is that in 82-kDa ChAT-expressing SH-SY5Y cells, 82-kDa ChAT and SATB1 localized to S/MARs and prevented an increase in an *APP* mRNA isoform that correlates with severity of cognitive impairment when cells were exposed acutely (4 h) to oligomeric Aβ_1–42_. Mastroeni et al. (2013) observed that longer (36 h) exposure to oligomeric Aβ_1–42_ resulted in inappropriate cytoplasmic localization of DNMT and RNA Pol II. Sen et al. (2015) found that, in addition to ApoE4, oligomeric Aβ_1–42_ increased the nuclear import of HDAC6 and HDAC4 in SH-SY5Y and human neurons in primary culture. The authors showed that ApoE4 treatment further increased the import of HDAC6 above the levels of Aβ_1–42_ alone, while ApoE3 treatment reduced the effect of Aβ_1–42_ on both HDAC6 and HDAC4. These data lead to the intriguing hypothesis that these chromatin and epigenetic modifiers are neuroprotective by altering chromatin structure and transcriptional activation in response to acute Aβ_1–42_ stress. Chromatin organizers and epigenetic modifiers become mislocalized after long-term exposure to Aβ_1–42_, such as in MCI and AD, leading to abnormal activation or repression of transcription (Figure 3). In support of this model, Lu et al. (2014) found that while REST is mislocalized to autophagosomes in AD, the protein is neuroprotective during normal aging. Compared to wild-type mice, cortical cultures from REST knockout mice have significantly higher induction of pro-apoptotic genes following 8 h of exposure to oligomeric Aβ_1–42_, and a *C*.* elegans* strain with mutations of the REST homolog *spr-4* showed accelerated degeneration of glutamatergic neurons compared to wild-type worms in response to a stably integrated Aβ_1–42_ transgene (Lu et al., 2014).

The mechanisms that lead to the changes in localization of chromatin organizers in AD are not fully understood, but Mastroeni et al. (2013) showed that the redistribution of DNMT and Pol II was due to reduced nuclear import caused by decreased Ran-GTP mRNA and protein expression in both AD patients and in SK-N-Be(2) cells. In addition, Lee et al. (2006) have shown that importin-α is abnormally localized to Hirano bodies (intracellular aggregates of actin) in hippocampal neurons from AD patients. Both Ran-GTP and importin-α are part of critical nuclear import/export mechanisms that regulate the import of most nuclear proteins (reviewed in Chook and Blobel, 2001). Importantly, mutant huntingtin aggregates in Huntington’s disease patients leads to mislocalization and aggregation of nuclear pores, which results in defective nucleocytoplasmic transport (Grima et al., 2017). Disruption of the nuclear import and export mechanisms through polyglutamine expansion and aggregation of huntingtin leads to an impairment in nuclear envelope integrity, destabilization of nuclear compartmentalization and DNA damage (Gasset-Rosa et al., 2017; Grima et al., 2017). Thus, targeting nuclear import and export mechanisms could be an attractive target for future AD interventions, to either re-establish nuclear levels or prevent the inappropriate redistribution of chromatin organizers in AD.

Another important approach for many aging-related diseases (e.g., Parkinson’s disease, Huntington’s disease) is to identify susceptibility genes as therapeutic candidates. Despite efforts to identify risk alleles and SNPs associated with AD, only the *APOE ε4* allele has been accepted as a susceptibility gene, with between 5 and approximately 20 other potential candidates (Bertram, 2011; Lambert et al., 2013; Altmann et al., 2014; Chen et al., 2016; Giri et al., 2016). As mentioned previously, this is in stark contrast with the hundreds of genes with altered gene expression in AD patients but not in aging individuals (Miller et al., 2008). If the transcriptional changes observed in AD are due, at least in part, to epigenetic or chromatin topology changes because of mislocalized proteins, then a few important questions arise: what are the risk factors for the changes in nuclear localization of these chromatin modifiers, and why are some individuals more at risk than others? One way to begin to unravel this question would be to determine whole-genome architecture (e.g., using chromatin conformation capture techniques) to identify changes in contacting loci from necropsy tissue of individuals with MCI or sporadic AD, and compare those to age-matched neurologically-normal controls. Newer technologies, such as Hi-C (Lieberman-Aiden et al., 2009; Dixon et al., 2012; Fraser et al., 2015), have been employed to understand genome architecture in neuronal cell models or in dissociated tissue (Dixon et al., 2015; Fraser et al., 2015; Won et al., 2016; Bonev et al., 2017), but have not been used to explore genome structure in cells from their native environment. Recently, Beagrie et al. (2017) developed a new approach, genome architecture mapping (GAM), to determine whole genome architecture directly *in situ*. As the authors showed that GAM can be applied to frozen or fixed tissue, GAM could also be applied in principle to post-mortem patient tissues (Beagrie et al., 2017; Elsner, 2017; Finn and Misteli, 2017). However, using *post hoc* approaches to understand genome architecture in brain may not fully reflect susceptibility to AD, as it is complicated by the chromatin-related changes that are occurring due to the mislocalized proteins. Longitudinal studies would be an ideal solution, though appropriate animal models for sporadic AD are not well developed. Forny-Germano et al. (2014) showed that intracerebroventricular injection of oligomeric Aβ in a small group of macaques induces a sporadic AD-like phenotype, which is a promising model for future study.

Current pharmacological interventions for AD, such as cholinesterase inhibitors and the N-Methyl D-aspartate (NMDA) receptor antagonist memantine, have limited effectiveness while causing adverse side-effects in many patients (reviewed in Szeto and Lewis, 2016). Alternative therapeutic approaches aimed at reducing Aβ production or aggregation have had only limited success (Szeto and Lewis, 2016). Thus, despite the current limitations, an alternative approach targeting chromatin modifications and genome access should be considered for AD treatment. This type of strategy involves identifying topological states of chromatin that are relevant to disease risk, and then correcting this phenotype. For example, the clustered regularly interspaced short palindromic repeats (CRISPR)-CRISPR associated nuclease 9 (CRISPR-Cas9) genome editing system has been used to explore gene therapy potential for many diseases, including HIV (Ye et al., 2014), several cancers (reviewed by Xiao-Jie et al., 2015), cataracts in mouse (Wu et al., 2013) and muscular dystrophy (Long et al., 2014), among others. Importantly, a CRISPR-Cas9 phase I clinical trial is already underway in T-cells for metastatic non-small cell lung cancer (Sichuan University and Chengdu MedGenCell Co. Ltd., 2016). The CRISPR-Cas9 system is an attractive approach for altering transcriptional state, correcting enhancer-promoter interactions, or for allowing/disrupting the binding of chromatin organizers (Guo et al., 2015; Hilton et al., 2015; Vojta et al., 2016). The CRISPR-Cas9 approach may be an important treatment strategy for familial AD where there are single gene mutations in APP or PSEN1/PSEN2 (Tanzi, 2012; Wu et al., 2012). However, CRISPR-Cas9 and similar genome editing techniques will likely need significant developments before they could be applied to polygenic diseases and diseases where there are changes to non-coding regions, such as in sporadic AD. Despite the current limitations, with advances in the understanding of the genomic landscape in neurons and in AD approaches that target non-coding regulatory regions of the genome may become an attractve future treatment strategy.

## Conclusion

Recent advances in understanding the role of chromatin organizers in AD (Mastroeni et al., 2013, 2015; Lu et al., 2014; Winick-Ng et al., 2016) represent significant steps towards elucidating AD etiology and progression. By considering AD as a disease that involves the dysregulation of chromatin organizers and topology, we can begin to untangle the complex epigenetic changes that occur to better target the core genome architecture changes that lead to synaptic dysfunction and neurodegeneration. Studies are needed to address what topological chromatin changes are directly relevant to disease progression, as the four-dimensional structure of chromatin is critical for context-dependent regulation of gene expression. Several critical proteins related to chromatin organization and epigenetic modifications are mislocalized in AD (Gill et al., 2007; Mastroeni et al., 2013, 2015; Lu et al., 2014; Sen et al., 2015). Future efforts are needed to address how nuclear import and export mechanisms become impaired for these proteins, and to further clarify the consequences of inappropriate redistribution of chromatin organizers to genome architecture and AD progression. Finally, important questions remain as to what contributions aging or neuronal senescence may have towards the dysregulation of chromatin in AD, and whether strategies that target re-establishing proper whole-genome architecture and epigenetic modifications will be an effective AD treatment strategy.

## Author Contributions

WW-N and RJR contributed to the conception and design of this review, and edited and revised the manuscript. WW-N wrote the manuscript and drafted the figures. WW-N and RJR approve of the final version to be published, and agree to be accountable for all aspects of the work.

## Conflict of Interest Statement

The authors declare that the research was conducted in the absence of any commercial or financial relationships that could be construed as a potential conflict of interest.

## Abbreviations

82-kDa ChAT: 82-kDa choline acetyltransferase
Aβ: β-amyloid peptides
AD: Alzheimer’s disease
APP: Amyloid precursor protein
BACE1: β-secretase 1
BDNF: Brain-derived neurotropic factor
BoE: Boundary element
CRISPR: Clustered regularly interspaced short palindromic repeats
CRISPR-Cas9: CRISPR associated nuclease 9
DNMT1: DNA methyltransferase 1
GAM: Genome architecture mapping
H3K27ac: Histone 3 lysine 27 acetylation
H3K27me3: Histone 3 lysine 27 tri-methylation
H3K4me3: Histone 3 lysine 4 tri-methylation
H3K9me3: Histone 3 lysine 9 tri-methylation
HAT: histone acetyltransferase
HDAC: histone deacetylase
LAD: Lamina associated domain
MCI: mild cognitive impairment
OR: Olfactory receptor
Pol II: RNA polymerase II
PSEN1/PSEN2: Presenilin 1/2
REST: Repressor element 1-silencing transcription
S/MAR: Scaffolding/matrix attachment region
SATB1: Special AT-rich binding protein 1
SMAR1: S/MAR-binding protein 1
TAD: Topologically associating domain.
